# Topics of Public Interest

**Published:** 1912-01

**Authors:** 


### TOPICS OF PUBLIC INTEREST

DR. W. W. KEEN of Philadelphia, has sent out a letter with copy of a petition signed by many medical men all over the country, including many with military experience, endorsing House Bill No. 30 for the reestablishment of the canteen. Members of the profession are requested to write to their senators and representatives (U. S., not state) and to Honorable John Hay, chairman of the House Committee on Military Affairs. This matter has been so thoroughly threshed out in the medical press that we do not need to repeat the arguments as to genuine temperance, diminution of venereal disease, increase of military efficiency and the saving of crime, due to the Canteen.

THE annual meeting of the Medical Society of the County of Chautauqua was held in Dunkirk, December 13, 1911. The following officers were elected: President, Dr. A. H. Eastman of Jamestown, Vice presidents, Drs. N. G. Richmond of Fredonia and George F. Smith of Falconer; Secretary-Treasurer, Dr. J. W. Morris of Jamestown.

---

EARLY in December, a resident of Gainesville, attending a funeral in Perry, developed suspicious symtoms and, on examination by local physicians and by Dr. Edward Clark of Buffalo, was decided to have small pox. He is now quarantined at his home in Gainesville.

---

LACKAWANNA is considering the establishment of a municipal telephone service. This is a wise form of public enterprise, which is the rule in Europe and whose general adoption in this country would result in a material reduction of prices and improvement of service, especially in the way of inter-urban connection. Conditions are much better than when we had to pay 14 cents a message on annual contract but the prices are still approximately 100 per cent. higher than in Europe.

---

THE Reverend Mr. Richeson of Boston, who is awaiting trial in connection with the death of his former fiancee by potassium cyanid, cudahied himself Dec. 19, probably taking for his text Matthew 5, 27-30. Self-mutilation of this kind is sufficiently common to constitute a type and is by no means characterized by coincident insanity, unless in an inclusive sense.

---

THE Tonawanda water works reports a pumpage of 136 million gallons for November, 1911, at a cost of 120 tons of coal. The water consumption was at the rate of 541 gallons per capita. As 100 gallons is considered a fairly adequate supply by many cities, it may be worth while to explain that Tonawanda has a large commercial demand for water.

---

DURING November, the Buffalo water works pumped 3,710 million gallons of water at a cost of 2,787 tons of coal. This represents about 1 1-3 million gallons per ton, as compared with 1 1-7 for Tonawanda. The per capita consumption was about 287 per capita, per diem. This is very high. It is worth noting that, the larger the city, the less the necessary per capita consumption of water, for several reasons: Opportunity for more careful inspection against waste; Relatively less area for street sprinkling, &c; Relatively less use for fire, on account of greater proportion of fire proof buildings; Relatively less use for commercial purposes, high land values driving large plants to smaller towns.

Le Roy will have medical inspection of its schools. Good. The children of small towns are just as valuable as those of large cities. Let other towns and villages follow Le Roy's example.

---

Mount Morris has lately voted down a proposition to obtain water from Silver Lake—and on a light vote at that.

---

The Elmira Academy of Medicine held its annual meeting Dec. 6, 1911. Officers were elected as follows: President, Dr. Herbert W. Fudge; Vice-President, Dr. John C. O'Brien; Secretary, Dr. Charles Haase; Treasurer, Dr. C. G. R. Jennings; Censors, Drs. A. J. Westlake, Elliot T. Bush and J. A. Bennett; Trustees, Drs. Sherman Voorhees and Ross G. Loop.

---

THE American Jewish Committee (356 Second Ave., N. Y.) asks our cooperation in an agitation to secure a fulfillment of treaty rights and an abolition of a form of persecution of their race. As many of us know, an ordinary European trip requires no passport. In fact, save for a few details, there is quite as much personal liberty in western Europe as in the United States, and vastly more so in regard to the right to purchase where one pleases and to hold his personal effects without official rummaging; vastly more so, too, in regard to personal violation of many moral and social obligations. Only in Bulgaria, Roumania, Turkey and Russia, is a passport necessary and only in one country in the world is the passport issued by the U. S. subject to local visé—in Russia. If the bearer of the passport states that he is a Jew or an atheist or if he refuses to answer as to his religion, he is denied entry. The Committee also claims, on investigation, to have found that the promises and representations of Russia, in reply to interrogation by the U. S. Government regarding the matter, have not been in good faith.

Whatever influence the Buffalo Medical Journal may have is strongly in favor of the broadest religious liberty and the fullest performance of treaty obligations. In one particular, however, we feel that the ultimate welfare both of our country and of citizens of other countries,, requires a sacrifice of a few private interests in the immediate future. No country should take a citizen or resident dependent of another country without the full consent of the latter and the discharge of all obligations to the parent country. It is the duty of this and other enlightened countries, not simply to afford an asylum for those lucky enough to escape from persecution but to compel, by their influence or by force, the essentials of religious and personal liberty under any government.

### Mortality Statistics in Cities of 100,000 Population and over in 1910.

### Advance Bulletin by the Census Bureau.

| Registration State. | Number of Deaths [1] from all causes per 1,000 Population. 1909 | 1910 |
|---|---|---|
| Total[2] | 14.2 | 14.7 |
| California | 13.4 | 13.5 |
| Colorado | 14.2 | 13.8 |
| Connecticut | 15.0 | 15.6 |
| Indiana | 12.9 | 13.5 |
| Maine | 15.6 | 17.1 |
| Maryland | 15.5 | 16.0 |
| Massachusetts | 15.4 | 16.1 |
| Michigan | 13.1 | 14.1 |
| Minnesota | ([3]) | 10.9 |
| Montana | ([3]) | 10.6 |
| New Hampshire | 16.9 | 17.3 |
| New Jersey | 14.7 | 15.5 |
| New York | 15.7 | 16.1 |
| North Carolina[4] | ([3]) | 18.7 |
| Ohio | 12.9 | 13.7 |
| Pennsylvania | 14.7 | 15.6 |
| Rhode Island | 15.6 | 17.1 |
| Utah | ([3]) | 10.8 |
| Vermont | 15.7 | 16.0 |
| Washington | 9.8 | 10.0 |
| Wisconsin | 11.8 | 12.0 |

1 Exclusive of stillbirths.
2 Includes District of Columbia.
3 Nonregistration state.
4 Includes only municipalities having a population of 1,000 or over in 1900.

| Birth Registration States. | Births, 1910. [1] | Deaths of infants under 1 year, 1910. [2] | Deaths of infants per 1,000 living births. 1910 [3] | 1909 | 1908 |
|---|---|---|---|---|---|
| Connecticut | 27,291 | 3,476 | 127 | 127 | 122 |
| Maine | 15,578 | 2,108 | 135 | 118 | 125 |
| Massachusetts | 86,765 | 11,377 | 131 | 125 | 130 |
| Michigan | 63,566 | 7,912 | 124 | 117 | 119 |
| New Hampshire | 9,385 | 1,373 | 146 | 154 | 146 |
| Pennsylvania | 202,631 | 28,377 | 140 | 131 | 138 |
| Rhode Island[4] | 6,595 | 1,111 | 168 | 142 | 132 |
| Vermont | 7,343 | 791 | 108 | 114 | 110 |

[1] Provisional and exclusive of stillbirths.
[2] Exclusive of stillbirths.
[3] Based on provisional figures for births.
[4] Exclusive of Pawtucket and Providence, R. I., for 1910, for which the returns were not received from the state board of health in time for inclusion.

| Registration City. | Death rates from all causes [1] per 1,000 Population. 1909 | 1910 |
|---|---|---|
| Birmingham, Ala | 18.2 | 19.5 |
| Los Angles, Cal | 13.7 | 14.0 |
| Oakland, Cal | 14.2 | 12.7 |
| San Francisco, Cal | 15.0 | 15.1 |
| Denver, Col | 17.0 | 16.4 |
| Bridgeport, Conn | 14.4 | 15.2 |
| New Haven, Conn | 16.9 | 16.5 |
| Washington, D. C. | 19.0 | 19.6 |
| Atlanta, Ga | 17.2 | 18.9 |
| Chicago, Ill | 14.6 | 15.1 |
| Indianapolis, Ind | 14.3 | 16.3 |
| Louisville, Ky | 15.5 | 16.7 |
| New Orleans, La | 20.2 | 21.3 |
| Baltimore, Md | 18.7 | 19.2 |
| Boston, Mass | 16.8 | 17.2 |
| Cambridge, Mass | 14.7 | 15.0 |
| Fall River, Mass | 19.1 | 18.4 |
| Lowell, Mass | 18.0 | 19.7 |
| Worcester, Mass | 15.5 | 16.9 |
| Detroit, Mich | 14.0 | 15.9 |
| Grand Rapids, Mich | 11.9 | 14.6 |
| Minneapolis, Minn | 10.7 | 12.3 |
| St. Paul, Minn | 11.4 | 11.9 |
| Kansas City, Mo | 14.4 | 15.9 |
| St. Louis, Mo | 15.8 | 15.8 |
| Omaha, Neb | 14.7 | 15.1 |
| Jersey City, N. J. | 16.8 | 16.3 |
| Newark, N. J. | 16.5 | 16.5 |
| Paterson, N. J. | 15.3 | 14.7 |
| Albany, N. Y. | 17.6 | 19.4 |
| Buffalo, N. Y. | 15.2 | 16.3 |

| | | |
|---|---|---|
| New York, N. Y. | 16.0 | 16.0 |
| Bronx Borough | 15.9 | 15.9 |
| Brooklyn Borough | 15.4 | 15.6 |
| Manhattan Borough | 16.6 | 16.5 |
| Queens Borough | 14.2 | 13.8 |
| Richmond Borough | 18.1 | 17.0 |
| Rochester, N. Y. | 14.4 | 14.6 |
| Syracuse, N. Y. | 14.5 | 15.4 |
| Cincinnati, Ohio | 16.5 | 17.4 |
| Cleveland, Ohio | 12.9 | 14.3 |
| Columbus, Ohio | 14.0 | 15.4 |
| Dayton, Ohio | 15.4 | 14.8 |
| Toledo, Ohio | 14.6 | 14.6 |
| Portland, Oreg | 9.8 | 11.0 |
| Philadelphia, Pa | 16.4 | 17.4 |
| Pittsburgh, Pa | 15.8 | 17.9 |
| Scranton, Pa | 16.3 | 16.4 |
| Providence, R. I. | 16.1 | 17.7 |
| Memphis, Tenn | 20.1 | 21.4 |
| Nashville, Tenn | 18.1 | 18.7 |
| Richmond, Va | 20.7 | 22.6 |
| Seattle, Wash | 10.0 | 10.1 |
| Spokane, Wash | 12.6 | 13.0 |
| Milwaukee, Wis | 13.7 | 13.8 |

1 Exclusive of stillbirths.

